# Effects of aquatic exercise on physical function and fitness among people with spinal cord injury: A systematic review

**DOI:** 10.1097/MD.0000000000006328

**Published:** 2017-03-24

**Authors:** Chunxiao Li, Selina Khoo, Athirah Adnan

**Affiliations:** a Department of Health and Physical Education, The Education University of Hong Kong, Hong Kong, China; b Sports Centre, University of Malaya, Kuala Lumpur, Malaysia.

**Keywords:** aerobic fitness, hydrotherapy, research synthesis, spinal cord damage, water rehabilitation

## Abstract

**Objective:**

The aim of this review is to synthesize the evidence on the effects of aquatic exercise interventions on physical function and fitness among people with spinal cord injury.

**Data source:**

Six major databases were searched from inception till June 2015: MEDLINE, CINAHL, EMBASE, PsychInfo, SPORTDiscus, and Cochrane Center Register of Controlled Trials.

**Study appraisal and synthesis methods:**

Two reviewers independently rated methodological quality using the modified Downs and Black Scale and extracted and synthesized key findings (i.e., participant characteristics, study design, physical function and fitness outcomes, and adverse events).

**Results:**

Eight of 276 studies met the inclusion criteria, of which none showed high research quality. Four studies assessed physical function outcomes and 4 studies evaluated aerobic fitness as outcome measures. Significant improvements on these 2 outcomes were generally found. Other physical or fitness outcomes including body composition, muscular strength, and balance were rarely reported.

**Conclusions and implications of key findings:**

There is weak evidence supporting aquatic exercise training to improve physical function and aerobic fitness among adults with spinal cord injury. Suggestions for future research include reporting details of exercise interventions, evaluating other physical or fitness outcomes, and improving methodological quality.

## Abstract 2

Supplemental Digital Content is available in the text

## 1 Introduction

Various physical therapies have been used in the rehabilitation of people with spinal cord injury (SCI). Physical therapy often includes exercise therapy to improve muscle function (strength and endurance) and aerobic capacity to prevent or reduce secondary problems such as pain and fatigue.^[1]^ Aquatic therapy provides an alternative form of exercise. The aquatic environment makes it easier for people with SCI to move their limbs and execute motor skills that are not possible on dry land.^[2]^

Aquatic therapy has been used in different populations including for people with rheumatic disease,^[3,4]^ fibromyalgia,^[5]^ Parkinson disease,^[6]^ multiple sclerosis,^[7]^ cerebral palsy,^[8]^ and also SCI.^[9]^ Two Cochrane reviews have been published on the effects of aquatic exercise. One review evaluated the effects of aquatic exercise for osteoarthritis of the hip or knee.^[10]^ It was reported that there were small-to-moderate effects short-term on function and reduction in pain. A more recent review on the benefits of aquatic exercise for fibromyalgia^[11]^ reported low to moderate improvements in symptoms and fitness. No serious harm was reported in both reviews. However, as aquatic therapy can be costly,^[12]^ it is therefore essential to evaluate the effectiveness of physical interventions for people with SCI.^[13]^

Despite the potential benefits of aquatic exercise, evidence regarding its effectiveness as a therapy for improving physical function and fitness among people with SCI is unclear. Therefore, the aim of this systematic review is to summarize the evidence for the effects of aquatic exercise on physical function and fitness among people with SCI.

## 2 Methods

As a review protocol for this research is not published, the Preferred Reporting Items for Systematic Reviews and Meta-Analyses (PRISMA)^[14]^ guideline was used to conduct this review (see Appendix 1 ). Ethical approval was not required as subjects were not recruited in this study.

### 2.1 Eligibility criteria

The inclusion and exclusion criteria were as follows:

Type of participants: Trials that recruited participants with acute or chronic, complete or incomplete SCI were included. Animal studies and studies with participants without SCI, or a mixed sample without data specific on participants with SCI were excluded.

Type of interventions: Studies that employed an aquatic exercise as an intervention (e.g., swimming, underwater treadmill walking) were included. Studies that did not use aquatic exercise as (part of) the intervention were excluded (e.g., functional electrical stimulation evoked exercise, learning to swim, surgery).

Type of studies: Studies that used an intervention design were considered for inclusion. They may be randomized controlled trials, controlled trials, case series studies, or single case studies. Retrospective and prospective surveys were excluded.

Type of outcomes: Trials that reported the primary outcome on physical function (e.g., functional independence, muscle contraction, mobility, or walking ability) and the secondary outcome on physical fitness (e.g., aerobic or cardiovascular fitness, balance, body composition, muscular endurance, and strength) were included. Studies that reported outcomes other than physical function and fitness (e.g., spasticity, psychological wellbeing) were excluded.

### 2.2 Information sources and search

A systematic search was conducted for English language journal articles published until 30 June 2015 from the following databases: MEDLINE (1966–), CINAHL (1981–), EMBASE (exclusive of MEDLINE) (1947–), PsychInfo (1806–), SPORTDiscus (1830–), and Cochrane Center Register of Controlled Trials (1991–). Two groups of keywords were combined for the search: spinal cord injur∗, OR spinal cord lesion, OR spinal cord trauma, OR parapleg∗, OR quadripleg∗, OR tetrapleg∗; AND hydrotherapy, OR water exercise, OR aquatic exercise, OR water therapy, OR aquatic therapy, OR aquatic aerobics, OR water aerobics, OR aquatic physical therapy, OR swimming, OR swimming therapy, OR aquatic activity, OR water activity, OR water sport∗, OR water rehabilitation, OR aquatic rehabilitation, OR aquaerobics (see Appendix 2 for an example). A forward and backward search was conducted to identify additional articles. We also wrote to experts in this field to see whether they knew of additional articles on the topic.

### 2.3 Study selection

After removing duplicate articles, 2 reviewers (CL and SK) independently screened the remaining articles by reading the titles, abstracts, and if necessary full texts (when titles and abstracts provided inadequate information for determining whether studies should be included or excluded). Disagreements on study inclusion were resolved by discussion between CL and SK.

### 2.4 Data items and collection process

Both reviewers independently extracted the following data from the included studies: participant characteristics, including sample size, gender, age, injury level on the spinal cord, grade based on the American Spinal Cord Injury Association (ASIA) Impairment Scale, and time since injury, study design, intervention program, including type, length, frequency and intensity, outcomes related to physical function and fitness, effect sizes (Cohen's *d*), and adverse events. There were no disagreements regarding data extraction between the reviewers.

### 2.5 Risk of bias in individual studies

The modified Downs and Black Scale^[15,16]^ was used to independently assess research quality of the included studies. The scale is valid and reliable for assessing study quality of both randomized controlled trials and nonrandomized controlled trials such as cohort and case studies. There are 27 items assessing 5 broad categories in this scale (i.e., reporting, external validity, internal validity—bias, internal validity—confounding, and power). The overall score of the scale ranges from 0 to 28. A higher percentage of the maximum score indicates a higher quality study: <50% (weak), 50%–69% (fair), 70%–79% (good), and 80%–100% (very good).^[16,17]^ A third reviewer was involved to resolve disagreements between the 2 reviewers.

### 2.6 Summary measures and synthesis of results

Cohen's *d* effect size was used as the principal summary measure for the intervention effect. The effect sizes (between-group differences) were either extracted or calculated based on available information. Meta-analysis was not conducted in this review given the heterogeneous study characteristics, intervention programs, outcome measures, and most included studies did not have a comparison group.

## 3 Results

### 3.1 Study selection

**Figure 1:**
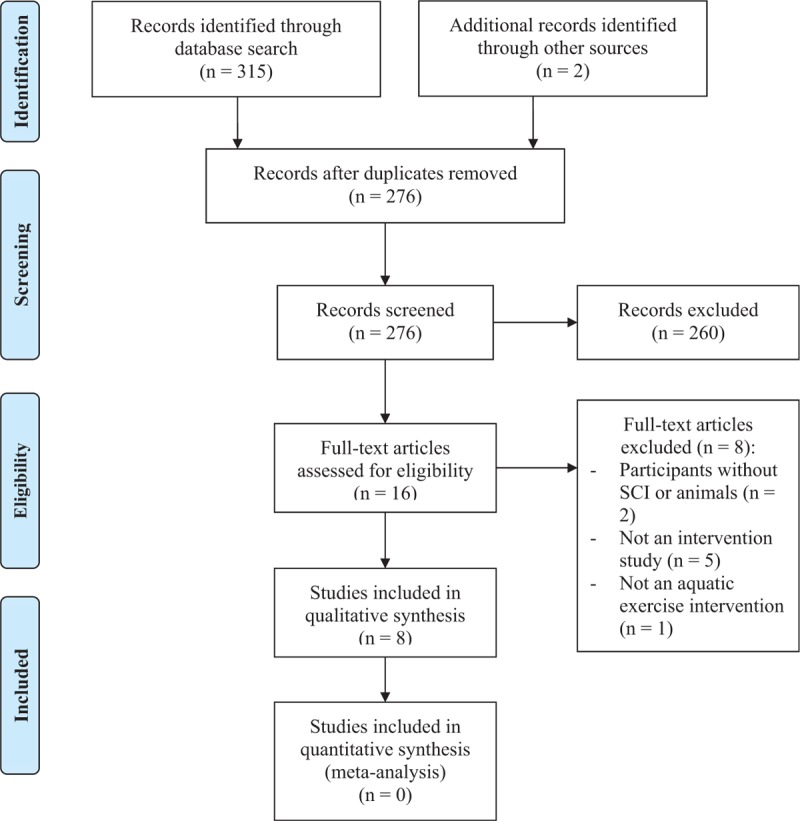
Process of study selection. SCI = spinal cord injury.

Figure 1 shows the process of study selection. The original search located 317 articles. After removing duplicates (i.e., overlapped articles across different databases), the remaining 276 articles were screened for eligibility through titles and abstracts. After screening, 260 articles were rejected: not participants with SCI (n = 20), not an intervention study (n = 153), not an aquatic exercise intervention (n = 65), not physical function or fitness outcomes (n = 20), and mixed participants without reporting data specific to the SCI group (n = 2). Full texts of the remaining 16 articles were read and 8 articles were excluded. Finally, 8 studies were included in this review.

### 3.2 Study characteristics

Table 1 presents the details of participants’ characteristics. A total of 143 participants with SCI were reported and the sample size of each study ranged from 1 to 60. More male participants were reported than female (male = 91, female = 52). Participants were adults aged between 18 and 63 years. Seven of 8 studies reported participants’ injury levels on the spinal cord (the study by Pachalski and Mekarski^[18]^ did not report the specific injury level). Only 4 studies provided the grade of ASIA impairment scale.^[9,19–21]^ There was a big range in terms of postinjury time from 7 months to 28 years. In terms of study design, 3 were controlled clinical trials,^[9,18,22]^ 2 single group test–retest designs,^[20,21]^ 1 randomized controlled trial,^[19]^ 1 single-subject design,^[23]^ and 1 case study.^[24]^

**Table 1:**
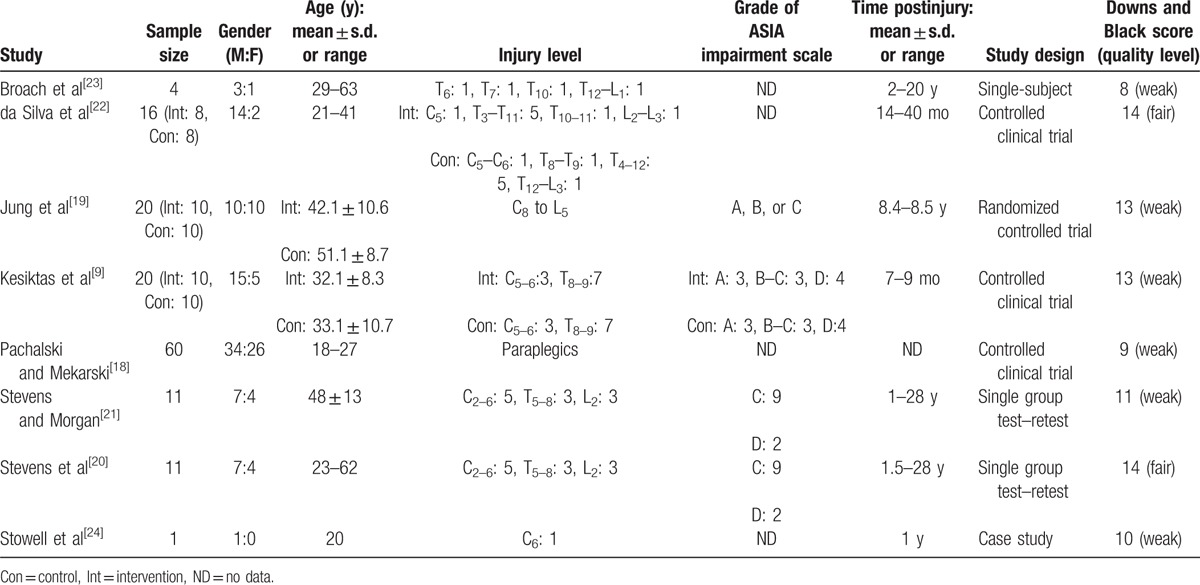
Participants’ characteristics and study design.

### 3.3 Interventions

Table 2 shows the details of intervention programs. Diverse intervention programs were used across the 8 included studies. One study used a swimming program^[23]^ and 2 studies incorporated swimming with physiotherapeutic activities as the intervention.^[18,22]^ One study used aquatic exercises,^[19]^ 1 study employed aquatic exercises together with land-based exercises,^[24]^ and 1 study combined aquatic exercises, physiotherapeutic activities, and oral baclofen as the treatment.^[9]^ Two studies utilized underwater treadmill training as their intervention program.^[20,21]^ Nearly all the intervention programs lasted between 8 and 16 weeks. One exception is the study by Pachalski and Mekarski,^[18]^ in which the intervention program lasted 3 years. Participants in all the included studies were required to participate in a training session 2 to 6 times per week with each session lasting between 20 and 60 minutes. Only 3 studies provided the information about training intensity, which ranged from low to high.^[18,20,21]^

**Table 2:**
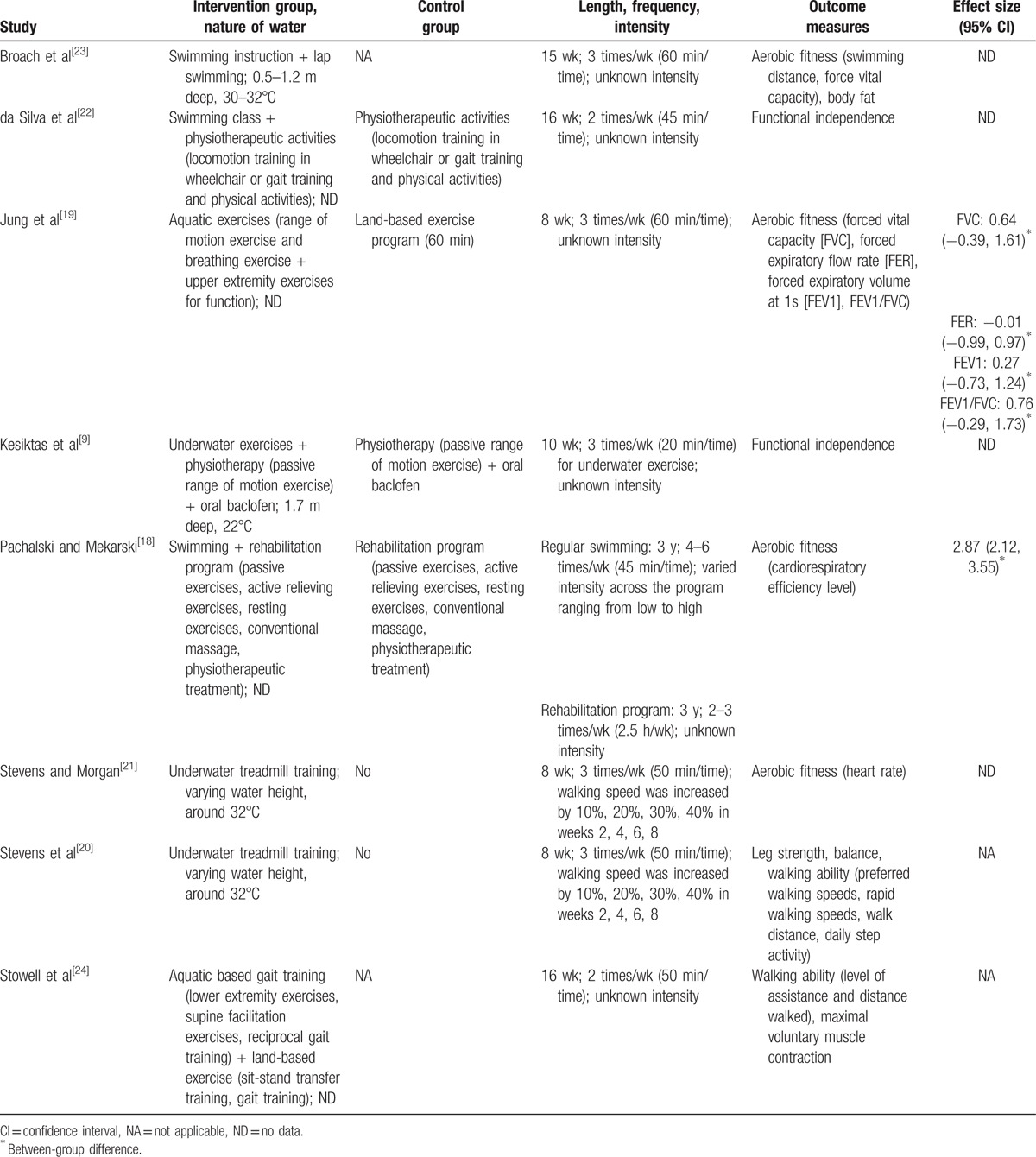
Interventions and outcomes.

### 3.4 Risk of bias within studies

The last column of Table 1 shows the total methodological scores of the included studies. Two studies^[20,22]^ showed fair quality and the rest^[9,18,19,21,23,24]^ were rated as weak research quality studies. Risk of bias was highest for power (0%), followed by external validity (4%), internal validity—confounding (23%), internal validity—bias (54%), and for reporting (63%).

### 3.5 Results of individual studies and synthesized findings

A variety of outcome measures were used in the studies (Table 2). As outlined above, meta-analysis could not be conducted in this review. Instead, qualitative synthesis of study outcomes was conducted. Namely, we aggregated their study findings if they reported the same outcome such as physical function.

### 3.6 Primary outcome: physical function

Physical function was reported in 4 studies.^[9,20,22,24]^ Compared with the physiotherapy control, participants’ functional independence was improved by either a 10-week program that combined aquatic exercises and physiotherapy (3 times/wk, 20 min/time for aquatic exercise session; effect size was not available),^[9]^ or a 16-week program combining swimming and physiotherapy (2 times/wk, 45 minutes; effect size was not available).^[22]^ In a within-subject test–retest study, an 8-week underwater walking program (3 times/wk, 50 min/time) showed improvement in participants’ walking ability (effect sizes were not available).^[20]^ Last, a case study with a 16-week aquatic and land-based exercise program was found to increase participants’ walking ability and their maximal voluntary muscle contraction (effect sizes were not available).^[24]^

### 3.7 Secondary outcome: physical fitness

Four studies reported outcomes related to aerobic fitness.^[18,19,21,23]^ A 15-week swimming program (3 times/wk, 60 min/time) was found to improve participants’ swimming distance and force vital capacity (FVC; effect sizes were not available) in a single-subject study.^[23]^ However, an 8-week aquatic exercise program (3 times/wk, 60 min/time) did not enhance participants’ FVC (effect size = 0.64), ratio between forced expiratory volume at 1 s and FVC (effect size = 0.76), forced expiratory flow rate (effect size = −0.01), and forced expiratory volume at 1 s (effect size = 0.27) when compared with the land-based exercise program.^[19]^ A 3-year program that combined swimming (4–6 times/wk, 45 min/time) and physiotherapy (2–3 times/wk, 2.5 hours/wk) was shown to largely benefit participants’ cardiorespiratory efficiency level (effect size = 2.87) compared with the physiotherapy control.^[18]^ Finally, an 8-week underwater treadmill walking program (3 times/wk, 50 min/time) decreased participants’ daily walking heart rate (effect size was not available) in a within-subject test–retest study.^[21]^

Only one study assessed outcomes related to body composition.^[23]^ In this single-subject study, participants’ body composition decreased through a 15-week swimming program (3 times/wk, 60 min/time; effect size was not available). Muscular fitness and balance were evaluated in only 1 within-subject test–retest study,^[20]^ in which participants’ leg strength and balance (effect sizes were not available) were increased after an 8-week underwater training (3 times/wk, 50 min/time).

### 3.8 Adverse events

Adverse events were only observed in 1 controlled clinical trial.^[9]^ Specifically, 2 participants in the control group that employed physiotherapy and oral baclofen experienced mild fatigue, which could be due to the use of oral baclofen.^[9]^

## 4 Discussion

To the best of our knowledge, this review is the first to systematically synthesize the evidence regarding the effects of aquatic exercise programs on physical function and fitness among people with SCI. An exhaustive search (see the Methods) found 8 studies that met the inclusion criteria. Overall, there is weak to fair evidence to support the effectiveness of aquatic exercise on the measured physical outcomes given the heterogeneous nature, low research quality, and synthesized results of the included studies.

### 4.1 Research quality

In regards to the research quality informed by the modified Downs and Black Scale scores, there was lack of power analysis among the included studies. Among the studies included in this review, only 1 was a randomized controlled trial, which may be due to the difficulty in using this research design in people with SCI (e.g., lack of access to the population, environmental barriers for the population to participate, and health conditions of the population). Due to the lack of high-quality randomized controlled trials, it is difficult to make definitive conclusions about the effectiveness of aquatic exercise on physical function and fitness among people with SCI. Half of the included studies did not have a control group, thus making the findings of the exercise program limited and providing weak evidence to support the effectiveness of the intervention. Another methodological concern is about blinding of therapists although it is difficult to achieve blinding in exercise interventions. It is impossible to blind participants.

### 4.2 Physical function

Participants’ physical functions were measured in 4 included studies with weak to fair methodological quality. Two^[9,22]^ assessed overall functional independence and the other 2^[20,24]^ evaluated walking ability. Despite the different measures across the 4 studies, aquatic exercise programs had a positive impact on physical function in all the 4 studies. This may be because the aquatic environment directly benefits and maximizes participants’ residual motor function, leading them to feel more independent in the aquatic environment after an adaptation period.^[9,22]^

### 4.3 Physical fitness

Four included studies with weak research quality evaluated the effect of aquatic exercise programs on aerobic fitness. Three studies^[18,21,23]^ showed a positive effect in improving participants’ aerobic fitness. Another study^[19]^ found that participants from the aquatic exercise group did not show better aerobic fitness than the land-based exercise group. However, within-subject differences were detected in the aquatic exercise group and there was a trend that the intervention may bring a larger effect in comparison to the land-based group. Despite the different measures across the 4 studies, these findings support the use of aquatic environments in improving aerobic fitness.^[25]^ According to the training programs of the 4 included studies, it seems that a training program that lasts for at least 8 weeks (3 times/wk, 50 min/time) would benefit participants’ cardiovascular fitness. Only 1 study^[23]^ with weak evidence investigated the effect of aquatic exercise on body composition, and another study^[20]^ with weak research quality assessed participants’ leg strength and balance. Therefore, there is minimal evidence in this review to draw conclusions on these 3 exercise outcomes.

### 4.4 Limitations

As this review was limited to journal articles published in English, we may have missed studies published in other forms (e.g., thesis) and languages. In addition, due to the heterogeneity of the included studies and most of them did not include a comparison group, it was inappropriate to conduct a quantitative synthesis. Finally, most of the included studies were low in methodological quality, which is likely to bias the review findings.

## 5 Conclusion

There is weak evidence supporting the effectiveness of aquatic exercise programs on improving physical function and aerobic fitness among people with SCI. It is premature to draw any conclusions about the effectiveness of aquatic training on body composition, muscular strength, and balance among the study population.

To provide further evidence about the effectiveness of aquatic exercise on physical function and fitness, more intervention studies should be conducted. It is necessary for future studies to include a control group instead of only relying on the within-subject differences. It would improve a study's research quality in particular to use power analysis, concealed allocation, and blinding assessors. Details of the intervention program such as training volume, exercise intensity, and spinal injury functional level should be clearly reported. This detailed information can be used to prepare related exercise guidelines in the future. Finally, in addition to physical function and aerobic fitness, it would be important to evaluate other physical fitness outcomes such as muscular strength and body composition.

## Supplementary Material

Supplemental Digital Content
