# Supplementary material for: Effects of aquatic exercise on physical function and fitness among people with spinal cord injury: A systematic review
Source: Medicine (Baltimore). 2017 Mar 24;96(11):e6328. doi: 10.1097/MD.0000000000006328 (PMC5369909; doi:10.1097/MD.0000000000006328)
Supplement: Supplemental Digital Content [file medi-96-e6328-s001.docx]

**Appendix 2.** Full Search Strategies for EMBASE

| **Step** | **Terms and qualifiers** |
| --- | --- |
| 1 | ((spinal cord injur* or spinal cord lesion or spinal cord trauma or parapleg* or quadripleg*) and tetrapleg*).af. |
| 2 | (spinal cord injur* or spinal cord lesion or spinal cord trauma or parapleg* or quadripleg* or tetrapleg*).af. |
| 3 | (hydrotherapy or water exercise or aquatic exercise or water therapy or aquatic therapy or aquatic aerobics or water aerobics or aquatic physical therapy or swimming or swimming therapy or aquatic activity or water activity or water sport* or water rehabilitation or aquatic rehabilitation, aquarobics).af. |
| 4 | (hydrotherapy or water exercise or aquatic exercise or water therapy or aquatic therapy or aquatic aerobics or water aerobics or aquatic physical therapy or swimming or swimming therapy or aquatic activity or water activity or water sport* or water rehabilitation or aquatic rehabilitation or aquarobics).af. |
| 5 | 2 and 4 |
| 6 | limit 5 to English language |
| 7 | limit 6 to (English language and exclude MEDLINE journals) |
